# Engineering human skin model innervated with itch sensory neuron‐like cells differentiated from induced pluripotent stem cells

**DOI:** 10.1002/btm2.10247

**Published:** 2021-08-20

**Authors:** Zongyou Guo, Chi‐Kun Tong, Joanna Jacków, Yanne S. Doucet, Hasan E. Abaci, Wangyong Zeng, Corey Hansen, Ryota Hayashi, Dominick DeLorenzo, Avina Rami, Alberto Pappalardo, Ellen A. Lumpkin, Angela M. Christiano

**Affiliations:** ^1^ Department of Dermatology Columbia University New York New York USA

**Keywords:** atopic dermatitis, innervation, iPSCs, itch; sensory neurons, skin

## Abstract

Atopic dermatitis (AD), driven by interleukins (IL‐4/IL‐13), is a chronic inflammatory skin disease characterized by intensive pruritus. However, it is unclear how immune signaling and sensory response pathways cross talk with each other. We differentiated itch sensory neuron‐like cells (ISNLCs) from iPSC lines. These ISNLCs displayed neural markers and action potentials and responded specifically to itch‐specific stimuli. These ISNLCs expressed receptors specific for IL‐4/IL‐13 and were activated directly by the two cytokines. We successfully innervated these ISNLCs into full thickness human skin constructs. These innervated skin grafts can be used in clinical applications such as wound healing. Moreover, the availability of such innervated skin models will be valuable to develop drugs to treat skin diseases such as AD.

## 1 INTRODUCTION

Human skin is a complex structure containing many cell types including sensory neurons, which mediate various sensation responses, such as pain and itch. Cutaneous sensory neurons have their cell bodies located in the dorsal root ganglia (DRG), with the nerve fibers branching into the dermis and moving upward into the epidermis.^1^ Sensory neurons are involved in neurologic disorders, immune responses and the regulation of aging and metabolism.^2^ Understanding the molecular mechanisms underlying sensation will benefit the development of strategies to treat skin diseases characterized by pain and itch.

Atopic dermatitis (AD) is the most common chronic skin disease, afflicting 2%–3% people in the adult population and 10%–30% in infants, thus causing a global disease burden.^3^ AD is both highly pruritic (itchy) and inflammatory,^4^ and skin inflammation can cause itch.^5^ Type 2 cytokines are well‐established mediators of skin inflammation in AD,^6^, ^7^ specifically the cytokine (IL‐4/IL‐13) driven AD pathogenesis.^8^ Notably, itch sensory neurons express receptors (IL‐4Rα and IL‐13Rα1) shared by IL‐4/IL‐13, and can be directly activated by the two type 2 cytokines,^9^ indicating a link between itch sensation and immune response. However, it is not known how the neuroimmune interactions are mediated by sensory neurons.

Human neurons are not readily available, which necessitates the use of animal models as surrogates.^10^ An organotypic skin model was developed that contained fibroblasts, keratinocytes, and neuronal cells,^11^ reminiscent of in vivo innervated skin with neurites branching through the dermis in proximity to keratinocytes. However, these neurons were derived from porcine DRG and the mechanisms of human nocioception and prunitoception are different from those of animals.^12^, ^13^ Several groups have attempted to circumvent the limited availability of human sensory neurons by conversion of fibroblasts to neurons using defined factors,^14^, ^15^ or by differentiation of human embryonic stem cells (hESCs) and induced pluripotent stem cells (iPSCs) to sensory neurons.^16^, ^17^, ^18^, ^19^, ^20^, ^21^, ^22^ However, no itch‐specific sensory neurons were produced. Thus, technical challenges in obtaining a sufficient number of sensory neurons precluded the development of a reliable model to investigate neuroimmune interactions with human cells.

To overcome this obstacle, here, we obtained iPSC‐derived sensory neurons (iNCs) which displayed action potentials, responded to a subset of itch‐specific stimuli, and demonstrated the ability to innervate human skin. The availability of functional itch‐specific iNCs will be of great value for delineating neuroimmune crosstalk as well as modeling AD to develop pharmaceutical agents.

## 2 RESULTS

### 2.1 Neurons differentiated from iPSCs exhibit molecular and morphological hallmarks of sensory neurons

**FIGURE 1:**
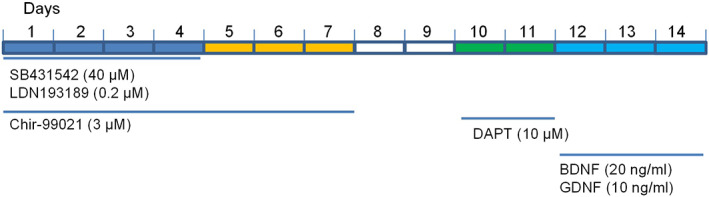
Schematic diagram of differentiation protocol. The treatment concentrations and durations are indicated except for days 8 and 9, when basal differentiation medium was used

**FIGURE 2:**
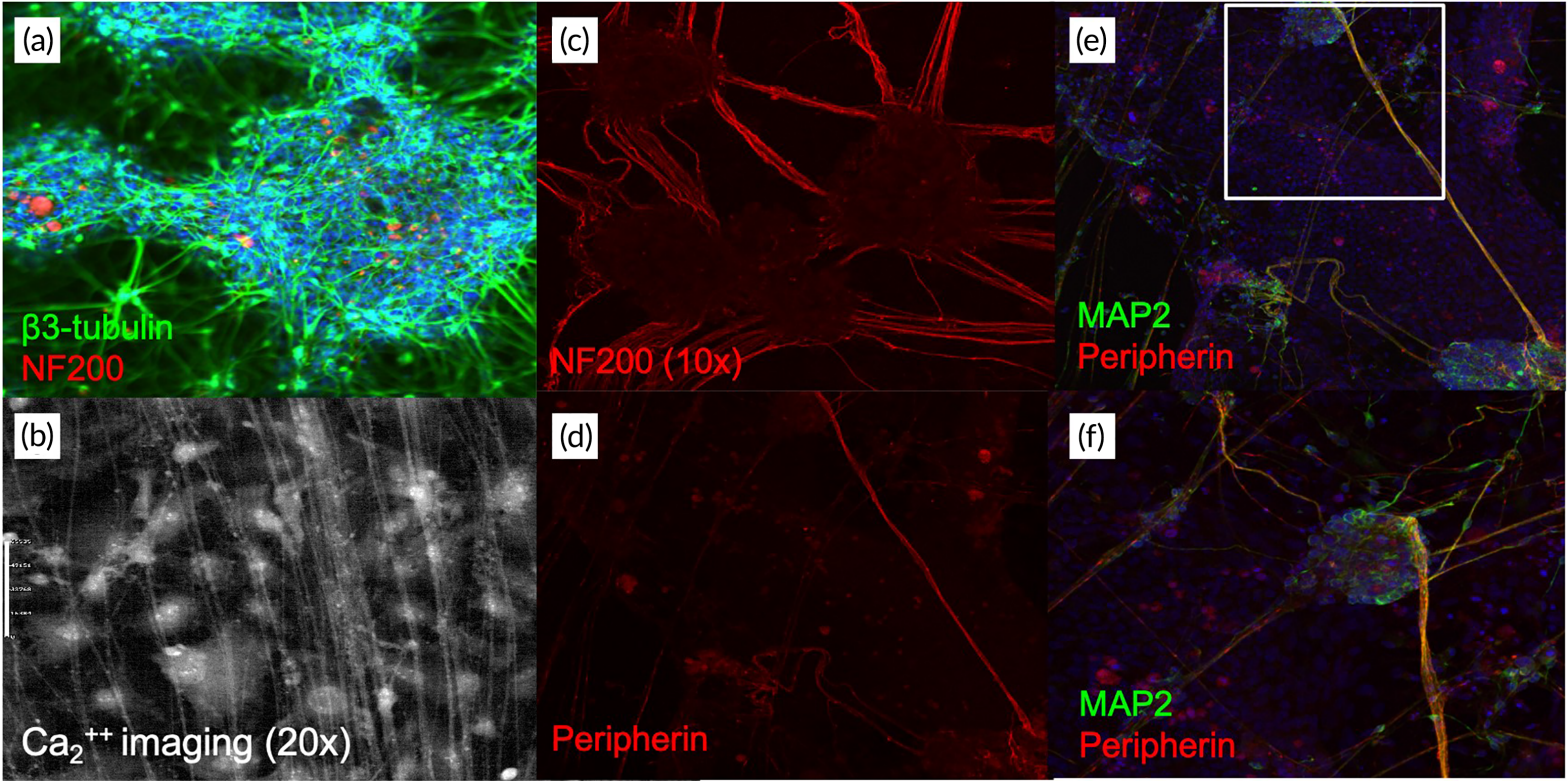
Immunological and morphological analysis of iNCs. iNCs were cultured on coverslips for Immunostaining (a and c–f) or morphological assessment (b). The antibodies used are indicated and DAPI (blue) staining is shown in figures a, e, and f. The morphological image was acquired with a differential interference contrast (DIC) microscope (b). The images were collected at a magnification of 10× (c–e), 20× (b and f), or 30× (a). Panel f is an enlargement of panel e (the white squares)

To obtain human sensory neurons, we sought to use iPSC technology because iPSCs have the potential to differentiate into many cell lineages, including sensory neurons. To this end, we obtained iPSCs using different metrologies, including a retroviral system,^23^ episomal vectors to reprogram human fibroblasts into integration‐free iPSCs,^24^ as well as feeder‐free culture conditions.^25^ Next, we screened different protocols to differentiate iPSCs into sensory neurons. A treatment with a high concentration (40 μM) of the transforming growth factor‐β (TGF‐β) inhibitor (SB431542) with 0.2 μM of bone morphogenetic protein (BMP) inhibitor (LDN 193189) for 4 days and 3 μM of Wnt agonist (Chir‐99021) for 7 days (Figure 1)^17^ gave rise to cells displaying the neuronal marker β_3_‐tubulin at day 14 (*N* = 3 dishes) (Figure 2a) and exhibiting neuron‐like morphology after culturing for additional 2 weeks (Figure 2b) (*N* = 6 dishes). Although the expression of NF20 (a marker for large myelinated A‐β fiber neurons) was low at day 14 after the initiation of differentiation (Figure 2a), its expression was increased in the mature neurites (Figure 2c) (*N* = 4 dishes).

The mature iNCs expressed peripherin (a type III Intermediate filament protein found mainly in median/small sensory neurons of the peripheral nervous system (PNS) (Figure 2d–f) (*N* = 4 dishes). Moreover, these cells displayed expression of microtubule‐associated protein 2 (MAP‐2) (a cytoskeletal protein marker for mature neurons) (Figure 2e,f) (*N* = 5). These data indicate that neurons differentiated from iPSCs express characteristic markers found in mature peripheral sensory neurons.

### 2.2 Human iNCs exhibit physiological properties of mature neurons

**FIGURE 3:**
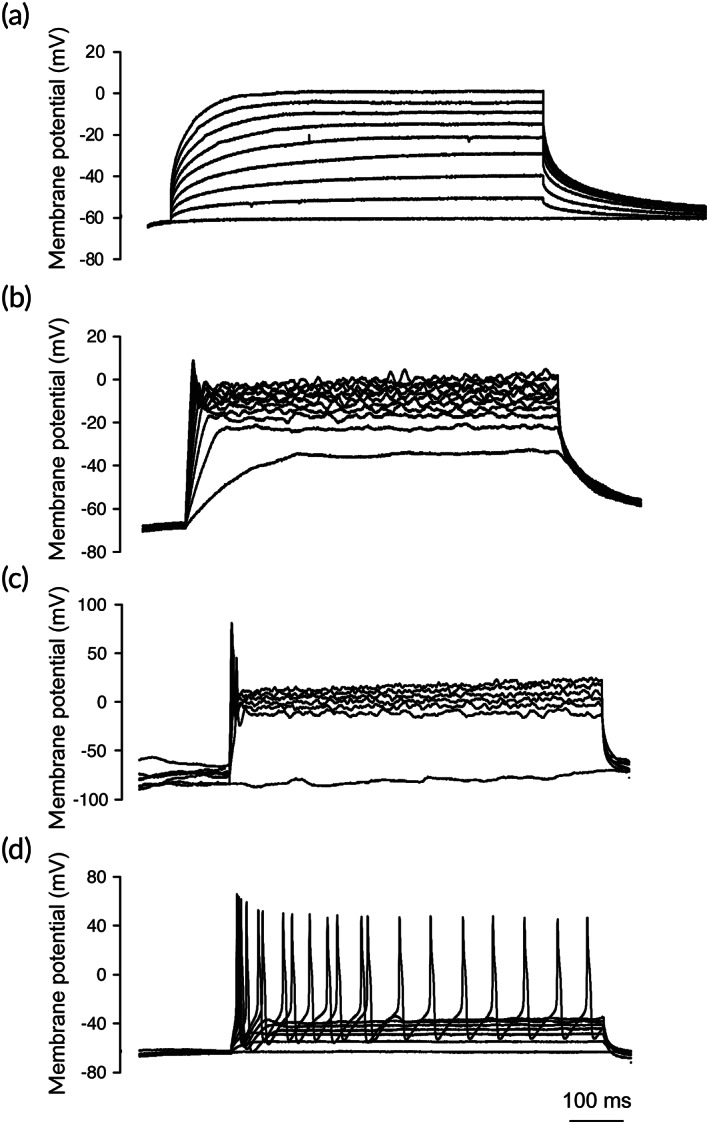
Whole cell patch‐clamp recordings of iNCs. iNCs were placed on coverslips and the current changes of the cells were recorded while the voltage remained constant. The cells were allowed to mature in a previously described protocol (a),^17^ modified media (b), or keratinocyte coculture (c and d). The modified media was formulated by using BrainPhis (Stem Cell Technologies)^26^ to replace the DMEM/F12 in the published medium^17^ and keratinocyte coculture was conducted in the modified medium for at least 2 weeks

Next, we performed whole cell patch‐clamp recordings to determine whether human iNCs were sufficiently mature to display the physiological properties (action potentials) of mature neurons. We found that the iNCs differentiated from our iPSCs with this method rarely showed any action potentials (Figure 3a) (= 6), suggesting that further optimization was needed.

Recently, a neuronal medium was developed to support activity of human neurons.^26^ We sought to improve the physiological properties with this approach by substituting the DMEM/F12 with this medium. Patch‐clamp recordings showed that some of the iNCs that were more matured with the modified medium but displayed no action potential or an abortive action potential (no action potential in 9 cells, abortive action potentials in 14 cells as shown in Figure 3b). These cells showed no response to high KCl, mustard oil, capsaicin, BAM8‐22, or menthol, but displayed small responses to chloroquine. Some of the cells showed strong response to histamine, mouse, and human PAR‐2, all of which are itch specific agonists (data not shown).

### 2.3 Keratinocyte coculture leads to physiological mature neurons

Sensory neurons reside in skin where many other cell types are also present in the environment and have close interactions with other types of cells (especially keratinocytes). We postulated that culturing iNCs with keratinocytes would enhance the functionality of the iNCs. We performed the coculture by plating iNCs on coverslips for 1 day and then placing the coverslips in dishes pre‐seeded with keratinocytes. Indeed, iNCs demonstrated single action potential firing (*n* = 3) (Figure 3c) or continuous firings (*n* = 1) (Figure 3d) after culturing with keratinocytes for 2 weeks. These results indicated that keratinocytes facilitate iNCs maturation to acquire the physiological properties of mature neurons.

### 2.4 iNCs are itch‐specific sensory neuron‐like cells

**FIGURE 4:**
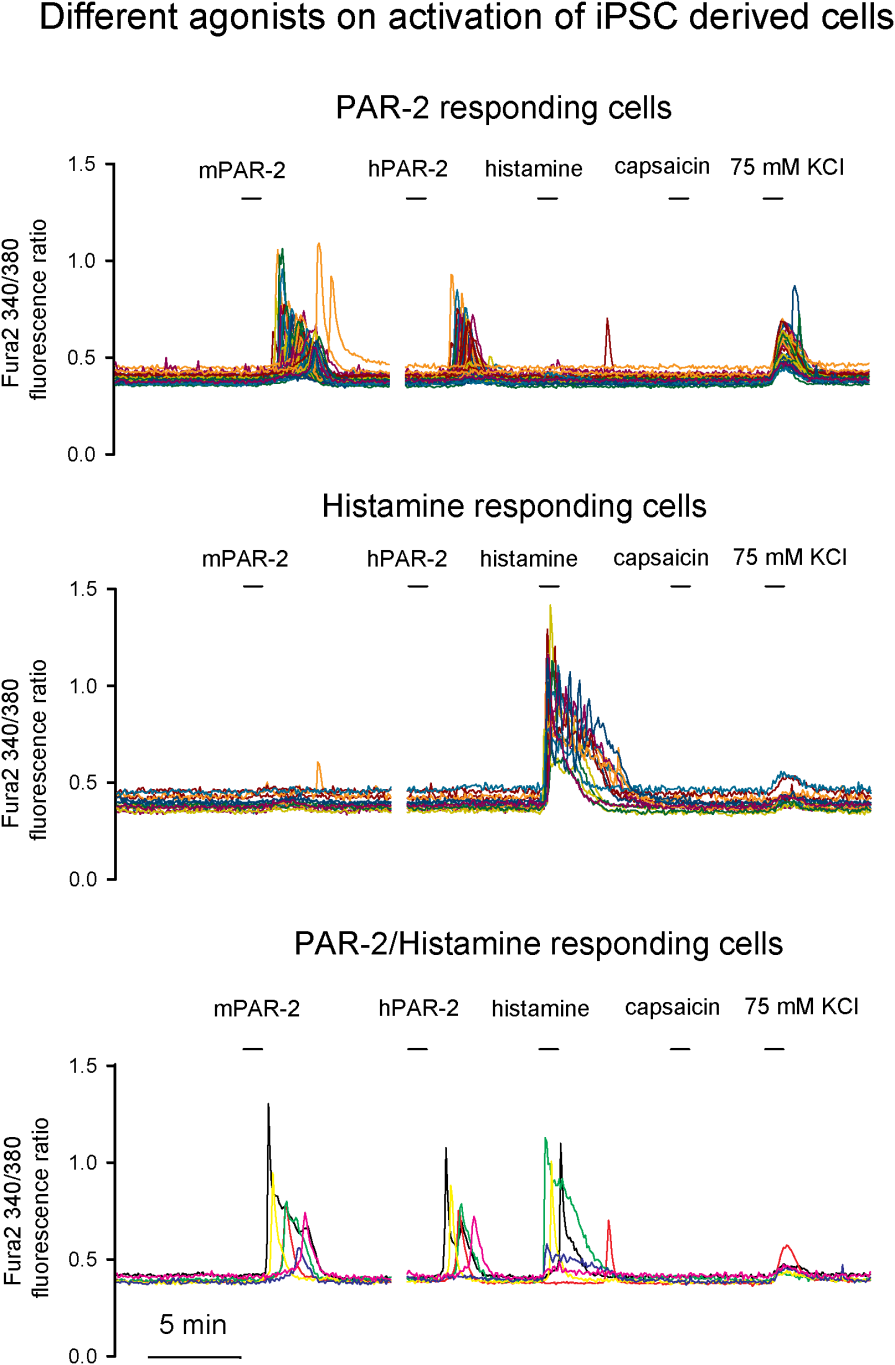
iNC calcium responses to sensational reagents. iNCs were treated with 10 μM of mouse (mPAR‐2) or human (hPAR‐2) protease‐activated receptor 2 peptides: SLIGRL‐NH_3_ (mPAR‐2) and SLIGKV‐NH_3_ (hPAR‐2). Also, histamine (100 μM) and capsaicin (1 μM) were administered at different time points as indicated and calcium responses were monitored by Fura2

Human sensory neurons have distinct subsets that mediate different sensory responses such as heat, cold, pain and itch. We tested whether the iNCs responded to non‐pruritogenic compounds (such as capsaicin, menthol, and mustard oil) as well as pruritogenes (such as histamine, chloroquine, the peptides BAM 8–22 and protease‐activated receptor 2 [PAR‐2] peptides). Two subsets of human iNCs responded selectively to histamine and PAR‐2 respectively (Figure 4). Notably, the iNCs responded to both human and mouse PAR‐2 peptides (Figure 4) (*N* = 4 dishes). However, the iNCs did not show responses to other chemical mimics for pain, heat (capsaicin, *N* = 5 dishes) and mustard oil (*N* = 2 dishes). These results indicate that the iNCs contains two itch‐specific subpopulations, responding to histamine (*n* = 23/114, 20%), PAR‐2 (*n* = 49/114, 43%), and both (*n* = 42/114, 37%, *N* = 2 dishes). Histamine is a potent inducer of pruritus and AD patients showed increased release of histamine in the skin. PAR‐2 is a member of protease‐activated receptors. Proteases like trypsin or tryptase can activate neuronal and/or keratinocyte‐derived PAR‐2, which subsequently leads to itch.^27^

### 2.5 iNCs migrate though the dermis toward the epidermis

**FIGURE 5:**
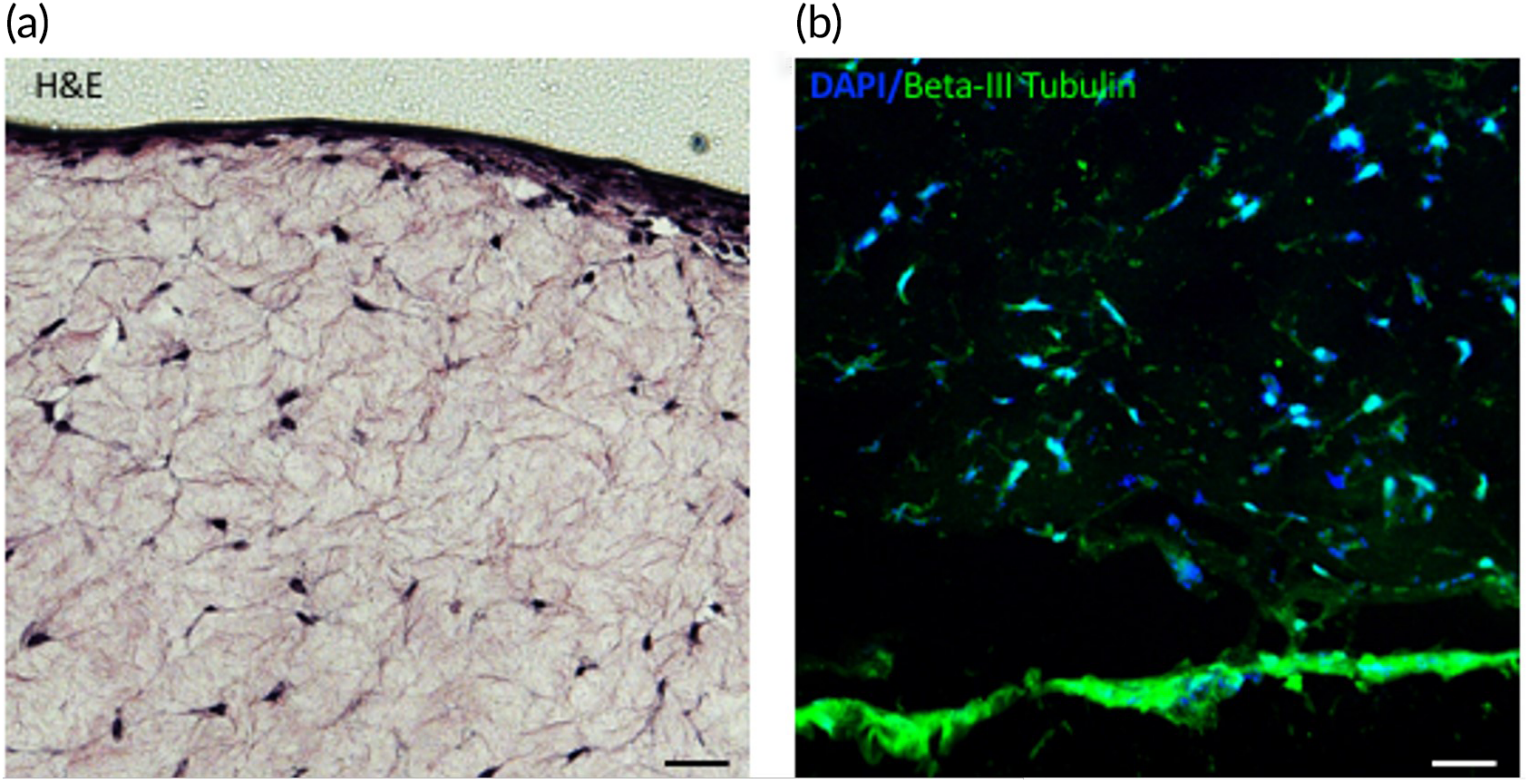
H&E and Immunostaining of innervated 3D skin constructs. The skin constructs were sectioned and analyzed by H&E (a) or immunochemical staining (b) (with antibodies as indicated). The images were acquired at a magnification of 20× (a and b). Scale bar: 100 μm

Human skin contains many types of innervated sensory neurons. We postulated that it might be possible to construct an innervated 3D skin model with our iPSC‐derived sensory neurons. To accomplish this, we dissociated iNCs and placed the cells beneath mature 3D constructs that were generated as we described previously,^28^, ^29^ containing fibroblasts in the dermis and keratinocytes in the epidermis. We placed iNCS outside the dermis and opposite to the epidermis. Two weeks after coculture, we observed neuron‐like cells in the dermis, indicating iNCs migrated into the dermal compartment toward the epidermis. The results showed that iNCs successfully migrated across the dermal compartment to reside in close contact of keratinocytes (Figure 5) (*N* = 6 dishes).

### 2.6 iNCs respond to IL‐4/IL‐13 treatment

**FIGURE 6:**
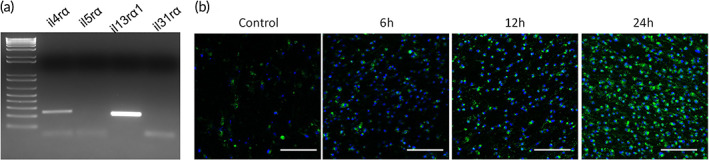
iNC expression of receptors for selected cytokines and calcium response to IL‐4/IL‐13 treatment. RNA was isolated from iNCs and subjected for RT‐PCR analysis (a) using published primer sequences^9^ (Table 2). Cells were treated with a combination of IL‐4/IL‐13 (20 μM/ml each) and imaged at different time points (as indicated) with a calcium indicator (Fura4) (b)

After verifying that the iNCs were physiologically reminiscent of itch sensory neurons, we next tested the potential of the cells to recapitulate features of AD. AD is an inflammatory disease of the skin (dermatitis), which causes the skin to itch, swell and crack. Recently, it was reported that thymic stromal lymphopoietin (TSLP), a keratinocyte‐secreted cytokine, can link inflammation with itch.^30^ In fact, both human and mouse DRG contain skin‐innervating sensory neurons, express IL‐4/IL‐13 specific receptors (*Il4rα* and *Il13rα1*), and can be directly activated by the two AD driver cytokines on itch‐sensory pathways.^9^ Therefore, we examined whether our itch‐specific iNCs would exhibit the same properties. We detected expression of *Il4rα* and *Il13rα1*, but not *Il5rα* (Figure 6a) (*N* = 3), which is similar to the expression pattern previously reported in cultured human DRG.^9^ However, we did not detect expression of *Il31rα* (Figure 6a), which is different from the previous results showing a faint PCR band,^9^ indicative of low expression in the cultured DRG. It is likely that *Il31rα* is only expressed in a subset of itch sensory neurons that were not present in our iNC population.

Having confirmed the expression of the receptors for IL‐4/IL‐13 in the iNCs, we utilized the cells to model AD, because these cytokines are key drivers of multiple atopic diseases including AD.^8^ We investigated whether these cytokines would elicit itch‐like responses in the iNCs in the absence of other cell types such as keratinocytes. We treated the iNCs with a combination of IL‐4/IL‐13, and the calcium responses were monitored with Fura4 at different time points. The results showed a time‐dependent response (Figure 6b) (*N* = 3), indicating that the AD driver cytokines IL‐4/IL‐13 can exert a direct effect on the sensory neurons, implying a potential utility of the iNCs in AD modeling and drug development.

## 3 DISCUSSION

We successfully differentiated human iPSCs into cells that recapitulated the morphology and characteristic markers of peripheral sensory neurons. These iPSC‐derived neurons (iNCs) fired action potentials and responded specifically to two types of chemical mimics of pruritogens, histamine and PAR2, indicative of functional properties of mature itch‐specific sensory neurons. These itch‐specific iNCs were responsive to AD driver cytokines (IL4/IL‐13) as indicated by calcium imaging.

The iNCs displayed the expression of the receptors shared by the AD driver cytokines (IL‐4/IL‐13) and could be directly activated to produce a calcium response after the treatment with the two cytokines, which agrees with previous findings.^9^ However, our RT‐PCR did not detect any expression of the receptor (*il31rα*). It is likely that *il31rα* is only expressed in a subset of sensory neurons that was not present in our iNCs, but in the DRG used for the previous study.^9^

Moreover, the iNCs are capable of innervating an in vitro 3D skin model. Recently sensory neurons were differentiated from iPSCs with a procedure similar to the one we used here. Their neurons were able to innervate 3D skin constructs, only in the presence of iPSC‐derived Schwann cells.^22^ Our iNCs had the ability to innervate 3D skin without the help of Schwann cells. It is likely that the subtle discrepancy in 3D skin construction and/or in iNC differentiation procedure between ours and the published protocol^22^ led to the difference in iNC innervation capability.

Protease‐activated receptors (PARs) are a group of four unique G‐protein‐coupled receptors. These receptors are self‐activated following specific proteolytic cleavage of their extracellular domains to expose a new amino terminus that acts as a ligand to activate PARs.^31^ Various extracellular proteases such as serine and cysteine proteases mediate PAR‐2 proteolysis. Microbial agents such as house dust mites, cockroaches, and bacteria secrete proteases to activate PAR‐2 and thus elicit itch.^27^ PAR‐2 activation likely stimulates itch pathways implicated in the pathophysiology of pruritus in AD.^32^, ^33^ Moreover, PAR‐2 antagonists and antibodies can alleviate itch‐related behavior in mice.^34^ These findings indicate that PAR‐2 may be a target molecule for the therapeutic treatment of AD.^35^ Our sensory neurons are responsive to PAR‐2 as well as to the AD driver cytokines (IL4/IL‐13). Organotypic skin models are responsive to IL‐4 and IL‐13,^36^ The in vivo dynamics of [Ca^2+^]_i_ in epidermal keratinocytes can be assessed with two‐photon microscopy.^37^ We postulate that IL‐4/IL‐13 treatment can perturb keratinocyte calcium dynamics. Thus, further IL‐4/IL‐13 experiments can be performed on organotypic skin models. AD skin displays increased innervation and pruritus is a prevalent symptom of the disease.^36^ Therefore, a 3D skin model innervated with itch‐specific sensory neurons would be advantageous to recapitulate AD pathophysiology and develop drugs to treat the disease. To this end, itch‐specific iNCs can be innervated into 3D skin and calcium imaging and/or AD‐specific markers can be used to assess AD pathogenesis induced by IL‐4/IL‐13 or Th2 cells. Drugs can be selected based on their ability to reduce AD markers on the innervated skin model. Thus, the availability of such cells will be valuable to model AD and develop therapeutic agents to treat the disease.

## 4 MATERIALS AND METHODS

### 4.1 Generation of iPS cells

We initially used a retroviral system to generate iPSCs with a feeder system.^23^ Then, to avoid the detrimental effect of random genomic integration by retrovirus, we evolved to Integration‐free iPSC reprogramming using the transfection of integration‐free episomal plasmids containing OCT3/4, SOX2, KLF4, and L‐Myc (Addgene plasmids 27077, 27078, 27080, 37624). Vectors were electroporated into fibroblasts with the Amaxa Nucleofector 2 Device (Lonza) with a NHDF Nucleofector kit.^24^, ^38^, ^39^ Recently, we combined our integration‐free iPSC reprograming methodology with a feeder‐free culture system to avoid the variability associated with feeders.^25^ The pluripotency of iPSCs was confirmed by immunostaining of stem cell markers and the capacity to form teratomas in nude mice.^23^, ^24^, ^25^

### 4.2 Neural differentiation

Sensory neurons were differentiated from iPSCs (Figure 1) based on the modification of a published protocol.^17^ Briefly, iPSCs were dissociated with accutase and seeded at 2 × 10^5^ cells/ml in 10‐cm petri dishes. Basal differentiation medium contained 1:1 of DMEM/F12 and Neurobasal (vol:vol), with N2, B27, β‐mercaptoethanol (0.1%), ascorbic acid (10 ng/ml) and Y‐27632 (5 μM). Differentiation was initiated by treatment with SB431542 (40 μM) and LDN 193189 (0.2 μM) for 4 days and Chir‐99021 (3 μM) for 7 days. On day 9, DAPT (10 μM) was included in the medium for 2 days. On day 11, embryoid bodies (EB) were dissociated into single cells with Trypsin 0.5% at 37°C for 10 min and plated on coverslips coated with poly‐ornithine (20 μg/ml) and laminin (5 μg/ml). From day 11, cells were allowed to mature in the presence of BDNF (20 ng/ml) and GDNF (10 ng/ml) in differentiation medium. In the event that some iPSC lines had difficulty with EB formation, differentiation was conducted until day 11 in AggreWell plates (Stem Cell Technologies) with 200–500 cells/microwell.

### 4.3 Calcium imaging

Coverslips containing attached iPSC‐derived cells was incorporated into a RC‐25 chamber (Warner Instrument, Harvard Apparatus) and incubated in Ringer's solution (145 NaCl, 5 KCl, 10 HEPES, 10 D‐Glucose, 2 MgCl_2_, 2 CaCl_2_, in mM, pH 7.3, osmolarity adjusted to 325 mmol kg^−1^ with sucrose) containing 5 μM fura‐2 AM (acetoxymethyl ester, Molecular Probes) and 0.02% pluronic F‐127 (Molecular Probes) for 30–45 min to allow fura‐2 AM to diffuse into the cells and convert to fura‐2. After washing out the extracellular fura‐2 AM with Ringer's solution, cells were imaged using an IX81 fluorescence invert microscope (Olympus). Olympus UApo 20X/0.75 objective and Chroma DM400, EM510/80 M, EX340, and EX480 filters were used to image the cells. Because fura‐2 is a ratiometric dye, its emission light intensity when excited at 340 nm was divided by that at 380 nm. The intensity ratio is positively correlated with cytoplasmic free calcium concentrations. Different agonists were perfused into the chamber for 30–60 s to test their reactivity with the cells. Agonists include: capsaicin (1 μM), menthol (250 μM), histamine (100 μM), mustard oil (250 μM), choloroquine (0.1 μM), ENMD (10 μM), BAM (8–22) (10 μM), mPAR‐2 (1–6) (10 μM), hPAR‐2 (10 μM), and HC‐067047 (10 μM) (Table 1).

**TABLE 1:** Sensation mimics

Reagents	Concentration (μM)	Sensation
Capsaicin	1	Pain
Menthol	250	Cold
Mustard oil	250	Heat
Chloroquine	0.1	Itch
BAM (8–22)	10	Itch/pain
mPAR‐2^a^	10	Itch
hPAR‐2^b^	10	Itch
Histamine	100	Itch

^a^ Mouse PAR‐2 peptides (SLIGRL‐NH_3_).

^b^ Human PAR‐2 peptides (SLIGKV‐NH_3_).

Time‐lapse images were collected and analyzed with MetaFluor for Olympus Software (7.5.6.0). Frame rates were 2–5 s. All experiments were performed at room temperature. KCl (75 mM) solution was perfused at the end of each experiment to identify if the cells were neurons.

### 4.4 Electrophysiology

Whole cell patch clamp recordings were done in the same Ringer solution. Intracellular solution was consisted of (in mM): K‐methylsulfonate 120, Na‐methylsulfonate 10, TEA·Cl 10, EGTA 10, CaCl_2_ 1, HEPES 10, Mg^2+^‐ATP 2.5, pH adjusted to 7.2 with KOH, osmolarity adjusted to 290 mOsm with sucrose. Junction potentials were measured and corrected in bath before the Gigaohm seal was formed for each cell. Axopatch 200B amplifier and pClamp 10.6 software were used to record the membrane potential under current clamp mode and analysis. Moderate depolarization of the membrane potential was used to test if the cell could fire an action potential. When a cell could not show clear activation of voltage‐gated sodium channel upon depolarizing to 0 mV, that cell was considered unable to fire action potentials. A membrane depolarization driven by the activation of voltage‐gated sodium channel, but failed to reach above 20 mV was considered an abortive action potential. All experiments were performed at room temperature.

### 4.5 RT PCR analysis

To determine the expression of cytokine receptors (*IL4Rα*, *IL5Rα*, *IL13Rα1*, and *IL31Rα*), RNA was purified with the RNeasy Mini kit (Qiagen), reverse‐transcribed into cDNA with the iScript™ Reverse Transcription kit (Bio‐Rad), and amplified with published primers^9^ (Table 2). RT‐PCR products were subjected to gel electrophoresis and visualized with SYBR Safe DNA Gel Stain (ThermoFisher) in 1% agarose gel.

**TABLE 2:** PCR primers for selected cytokine preceptors

Genes	Oligo sequences
*IL4Rα* RT‐PCR primer 1	GACGTGGTCAGTGCGGATAA
*IL4Rα* RT‐PCR primer 2	CTGAAATCTGCCGGGTCGTT
*IL5Rα* RT‐PCR primer 1	CTTGCGGTGCTTGTTAACGG
*IL5Rα* RT‐PCR primer 2	CGAGTGAACGGGTACGTTTCT
*IL13Rα1* RT‐PCR primer 1	CCTACGGAAACTCAGCCACC
*IL13Rα1* RT‐PCR primer 2	CCCCACTTGCAGACAAATCC
*IL31Rα* RT‐PCR primer 1	CACAAGAAAAGCTCGCAGACA
*IL31Rα* RT‐PCR primer 2	GGTGGTTCAGTTTTCGCTATGTT

### 4.6 Construction of innervated 3D skin models

3D skin was constructed following a previously described protocol.^25^ Briefly, a type I collagen matrix was used to embed normal human fibroblasts and the polymerized cell‐containing gel was incubated on polyethylene terephthalate membranes (BD Biosciences) for 5–7 days. Then, normal human keratinocytes were seeded onto the matrix, and incubated for additional 6 days, before the composite culture was raised to the air–liquid interface for 7 days to induce epidermal differentiation. For innervation, iNCs were seeded on a Matrigel‐coated transwell membrane before a skin construct was placed atop. Medium was fed from below for 2 weeks before the constructs were harvested for analysis.

### 4.7 Immunofluorescent imaging

For cultured iNCs, cells on cover slips were fixed with 4% paraformaldehyde at room temperature for 20 min before blocking with 1.5% fish skin gelatin (Sigma) in 0.1% Triton X‐100/phosphate‐buffered saline (PBS) for 1 h. Then, the fixed cells were incubated with primary antibodies at room temperature for additional 1 h. After rinsing with PBS, secondary antibodies were applied to the cells and incubated at room temperature for 1 h. VECTASHIELD Mounting Medium containing DAPI (Vector Laboratories) was used for nuclear staining before visualization with a confocal microscopy (Carl Zeiss LSM 5 EXCITER). All antibodies are listed in Table 3. For formalin‐fixed, paraffin wax‐embedded tissues, samples were sliced onto poly‐L‐lysine‐coated slides and dried overnight at 55°C. Subsequently, the slides were dewaxed in xylene and rehydrated through a graduated ethanol series (100%, 95%, 70%) and distilled water (dH_2_O) before antigen retrieval at 97°C in 10 mM sodium citrate buffer (pH 6.0) for 30 min. The blocking and incubations with primary and secondary antibodies were similar to the procedure described above.

**TABLE 3:** List of antibodies

Antibodies	Cat. No.	Vendor	Dilutions	Host
β3 tubulin	ab18207	Abcam	1:1000	Rabbit
NF200	ab72996	Abcam	1:25,000	Chicken
MRGPX2	ab188767	Abcam	1:250	Rabbit
Peripherin	sc‐7604	Santa Cruz Biotechnology	1:200	Goat
MAP2	4542S	Cell Signaling Technology	1:50	Rabbit

### 4.8 Hematoxylin and eosin staining (H&E)

After dewaxing as described above, sample slides were stained with Mayers Haematoxylin (Sigma) at RT for 3 min, followed by blue staining, rinsing in tap water, and differentiation with rinsing in 1% acid ethanol. Subsequently, the sections were counterstained by rinsing with eosin (Sigma) for 30 s and dehydrated by sequential washing with 95% ethanol, 100% ethanol and Histo‐Clear (National Diagnostics). After mounting with coverslips, slides were examined by light microscopy using a Zeiss Axioplan 2 microscope.

## AUTHOR CONTRIBUTIONS

**Chi‐Kun Tong:** Formal analysis. **Joanna Jacków:** Formal analysis; visualization. **Yanne Doucet:** Visualization. **Hasan Abaci:** Visualization. **Wangyong Zeng:** Data curation. **Corey Hansen:** Data curation. **Ryota Hayashi:** Data curation. **Dominick DeLorenzo:** Data curation. **Avina Rami:** Writing‐review & editing. **Alberto Pappalardo:** Data curation. **Ellen Lumpkin:** Supervision. **Angela Christiano:** Supervision; writing‐review & editing.

### PEER REVIEW

The peer review history for this article is available at https://publons.com/publon/10.1002/btm2.10247.

## Data Availability

Data available on request from the authors.
